# Signature and Molecular Mechanism of Mitochondrial Energy Metabolism Pathway-Related Genes in Lung Adenocarcinoma

**DOI:** 10.1155/2022/3201600

**Published:** 2022-08-22

**Authors:** Jiayuan Liu, Feng Zhang, Jinlong Zhong, Zhi Zheng

**Affiliations:** ^1^Department of Thoracic Surgery, Jinshan Hospital of Fudan University, Longhang Road No. 1508, Jinshan District, Shanghai 200540, China; ^2^Department of Cardiology, Jinshan Hospital of Fudan University, Longhang Road No. 1508, Jinshan District, Shanghai 200540, China

## Abstract

**Objective:**

The mitochondrial energy metabolic pathway (MEMP) is the primary energy metabolism of tumor cells, and its disruption may promote cancer emergence, spreading, and immune escape. However, there is a lack of studies to determine the relationship between relevant functional mechanisms and lung adenocarcinoma (LUAD) prognosis.

**Methods:**

Gene set enrichment analysis (GSEA) was employed to determine MEMP pathway-related genes. Then, a prognostic model was created using the MEMP key genes that were found by LASSO-Cox regression analysis. The Cancer Genome Atlas (TCGA) and Gene Expression Omnibus (GEO) databases provided the training and validation sets. Furthermore, the infiltration of immune cells was examined by ssGSEA. Finally, a screening of candidate therapeutic compounds for LUAD patients was performed using DrugBank, Protein Data Bank (PDB), and AutoDock Vina databases.

**Results:**

First, 266 MEMP pathway-related genes that exhibited aberrant activity in tumors were identified. Then, 19 MEMP key genes were used to build a prognostic model, which can successfully predict the survival rates of LUAD patients after 1, 3, and 5 years, respectively. The Kaplan-Meier curve showed that patients in the high-risk group had considerably lower survival outcomes than those in the low-risk group. Furthermore, it was discovered that the high-risk group had the majority of activated T cells, while the low-risk group tended to have more other activated immune cells. The majority of immunological checkpoints expressed themselves more strongly in the high-risk group as well. Finally, 11 prospective medication small molecules were obtained from the projected potential therapeutic drugs, with DB0980 being regarded as the most promising of them for the treatment of LUAD.

**Conclusion:**

This current study developed reliable prognostic signature, called MEMP score, which provides new guidance for prognostic assessment, immunotherapy, and drug development in LUAD. Thereby, DB0980 appears to be the most likely approach for the treatment of LUAD.

## 1. Introduction

According to the most recent statistics on cancer, there will be 28.4 million cases of the disease worldwide in 2040, and lung cancer will account for the majority of related deaths, outpacing all other cancer types by a wide margin. The major cause of death from cancer is still lung cancer [1]. The most common form of lung cancer, accounting for about 40% of cases, is lung adenocarcinoma (LUAD) [2]. LUAD incidence rates increase every year, especially among women and young people. The five-year survival rate of LUAD patients is less than 20% despite progress in diagnosis and treatment [3]. It is challenging to enhance the therapeutic effect due to a lack of knowledge of the underlying LUAD mechanism. Consequently, a prognostic signature must be created in order to increase the ability to correctly predict the prognosis of LUAD [4].

The energy metabolic pathways that tumor cells depend on for survival include glycolysis and mitochondrial oxidative phosphorylation. Abnormal mitochondrial-related pathways and metabolic disorders that affect gene expression can aid the genesis and progression of cancer, as well as the escape of immune system. Therefore, aberrant energy production may result from mutations and altered expression of genes connected to the mitochondrial energy metabolism pathway (MEMP). Studies have revealed that UBE4B can promote glycolysis, migration, and proliferation through the PP2A/AKT signaling, which can help generate LUAD [5]. Additionally, research has demonstrated that genes associated with glycolysis can effectively predict prognosis and reflect immunological status [6], revealing that genes associated with glycolysis play a crucial role in prognosis. The oxidative phosphorylation-related gene MTFR2, which has been proven in studies to influence prognosis in LUAD, can be employed as a potential prognostic indicator and therapeutic target for this disease [7]. Therefore, it is probable that the key genes involved in the mitochondrial energy metabolism system will influence lung adenocarcinoma prognosis and act as appropriate therapeutic targets.

This bioinformatics study was aimed at evaluating the role of the MEMP key genes in LUAD, at developing the MEMP signature as a new prognostic feature of LUAD, and at distinguishing between low-risk and high-risk LUAD patients to clarify the potential difference. Thereby, survival and tumor microenvironment were relevant issues of interest. In consequence, potential novel therapeutic drugs should be predicted and evaluated on the basis of molecular docking as a basis for future research.

## 2. Materials and Methods

### 2.1. Dataset Collection and Processing

The clinical information and expression data of LUAD were acquired from The Cancer Genome Atlas (TCGA, https://cancergenome.nih.gov/).There were 510 primary cancer samples and 58 paracancerous samples in expression profile data, and then, 497 tumor samples with both expression and survival information were retained for follow-up analysis. The clinical phenotypes of 497 patients with LUAD were rated, composed of age, gender, tumor stages, EGFR mutation, and EML4-ALK translocation (Table 1).

The validation set was collected using Gene Expression Omnibus (GEO; https://www.ncbi.nlm.nih.gov/geo/), and the GSE72094 and GSE42127 datasets were used for verification of the prediction model. The Molecular Signatures Database (MSigDB, http://www.gsea-msigdb.org/gsea/msigdb/) was used to find gene sets relevant to mitochondrial energy metabolism [8, 9]. The human gene transfer format (GTF) file was provided by the GENCODE database (https://www.gencodegenes.org/) [10]. The PDB database supplied the 3D structural information for proteins (https://www.rcsb.org/) and the DrugBank database (https://go.drugbank.com/) provided the information on chemical structures.

### 2.2. Gene Set Enrichment Analysis

Gene set enrichment analysis is a calculation technique used to ascertain whether a selected gene set has significant enrichment differences between tumors and adjacent tumors. Initially, using gene set enrichment analysis (GSEA), MEMP-related pathways with significant enrichment differences (*P* value < 0.05) were discovered in patients with tumor and paracancerous tissues. The key genes in the significant gene set were identified and aggregated as MEMP key genes for further study.

### 2.3. MEMP Key Gene Mutation Analysis

Based on the key gene of MEMP, the overall mutation of the gene was displayed through the mutation data of TCGA. The MAF-formatted mutation data were displayed and annotated using the R program maftools.

### 2.4. LASSO-Cox Regression Analysis and Construction of Prognostic Risk Model

Based on the TCGA-LUAD expression data, the univariate Cox analysis with a threshold of 0.05 was used to investigate the key MEMP genes. Categorical factors were used to specify the prognosis-related genes. To reduce the possibility of overfitting, a prognostic model was built using LASSO-Cox regression analysis. According to the pathway genes' expression levels and matching regression coefficients, the scores of the patients were determined:

(1) $$\begin{array}{c} \text{Score}=\overset{n}{\underset{i=0}{\sum }}\beta \text{i}*\chi \text{i}, \end{array}$$

where *β*i is the weight coefficient of each gene and *χ*i is the expression of each gene.

LUAD patients were divided into high-risk and low-risk groups based on a score derived from survival time, patient status, and TCGA-LUAD expression data. Each model gene was examined by the Kaplan-Meier survival analysis (R package survminer) based on log rank. Univariate and multivariate analyses utilizing the Cox regression were combined with other clinical variables to ascertain the independent predictive value of scores. The predictive power of prognostic characteristics was assessed using ROC curve analysis (R package timeROC). A statistically significant *P* value was 0.05. In addition, the GSE72094 and GSE42127 datasets were used for verification.

### 2.5. Subgroup Analysis and Construction of Nomograph

The patients were divided into subgroups according to different clinicopathological features. In order to examine statistical variances in risk ratings, the Wilcoxon test was utilized between two groups, and the Kruskal-Wallis test was used in multiple groups. All independent prognostic variables in the multivariate Cox regression analysis were utilized to generate a nomogram incorporating clinical characteristics and risk scores using R-package RMS to combine the outcomes of the risk-scoring model with clinical qualities. Then, calibration curves were used to assess the precision of nomograms in predicting 1-, 2-, and 3-year survival in LUAD patients.

### 2.6. Correlation Analysis of Immune Cell Infiltration and Evaluation of Immunotherapy Effect

The ssGSEA estimated and visually interpreted the Pearson correlation between the model gene and the immune cell infiltration score [11]. TCGA-LUAD transcriptome data and high-risk and low-risk score groups were used to analyze the differences in immune checkpoint expression [12] between high-risk and low-risk score groups.

### 2.7. Screening of Potential Therapeutic Compounds for LUAD

According to Lipinski's criteria, the structural data of the corresponding compounds downloaded from the DrugBank database were screened. The spatial structure information from the PDB database for the top six signature genes of prognostic significance, ERO1A, FKBP4, PKP2, PPIA, RPE, and VDAC1, was obtained, and the accompanying PDB files, 3AHQ, 4LAX, 3TT9, 5TA2, 3OVR, and 5XDO, were downloaded. For docking with small molecules, AutoDock Vina was utilized, and Pymol and Ligplus were employed for interaction analysis.

## 3. Results

### 3.1. GSEA to Select the Key Genes of MEMP

**Figure 1:**
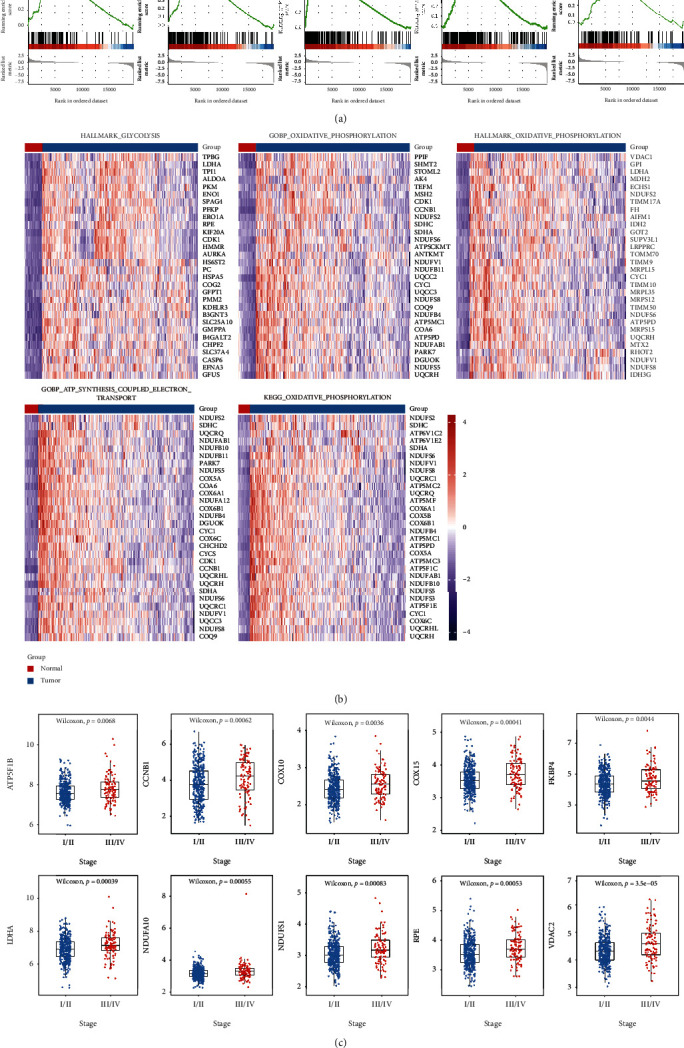
Key genes of MEMP. (a) Significant mitochondrial energy metabolism-related pathways. (b) The difference in key gene between tumor and control. (c) Distribution of the expression of MEMP top 8 genes.

Five gene sets were found to be significantly activated (*P* value < 0.05) in LUAD using GSEA analysis in 22 gene sets of mitochondrial energy metabolism through MSigDB. These gene sets included glycolysis (1), ATP electron transport chain (1), and oxidative phosphorylation (3) (Figure 1(a)). The key genes of MEMP were then combined as the key genes of the five pathways, which comprised 266 genes in total (Table 2). MEMP key genes were highly expressed in tumor tissues, as shown by the heat map, which compared the expression of each gene sets top 30 key genes in tumor tissues to controls (Figure 1(b)). The majority of the key genes in TCGA-LUAD showed significant differences in expression between stage I/II and III/IV (*P* value < 0.05), and they were highly expressed in stage III/IV. The top two differentially expressed genes in each gene set were significantly expressed in tumor tissues and the *P* value was far below 0.05, indicating that MEMP was dysfunctional in LUAD (Figure 1(c)).

### 3.2. Epigenetic Analysis of Key Genes of MEMP

**Figure 2:**
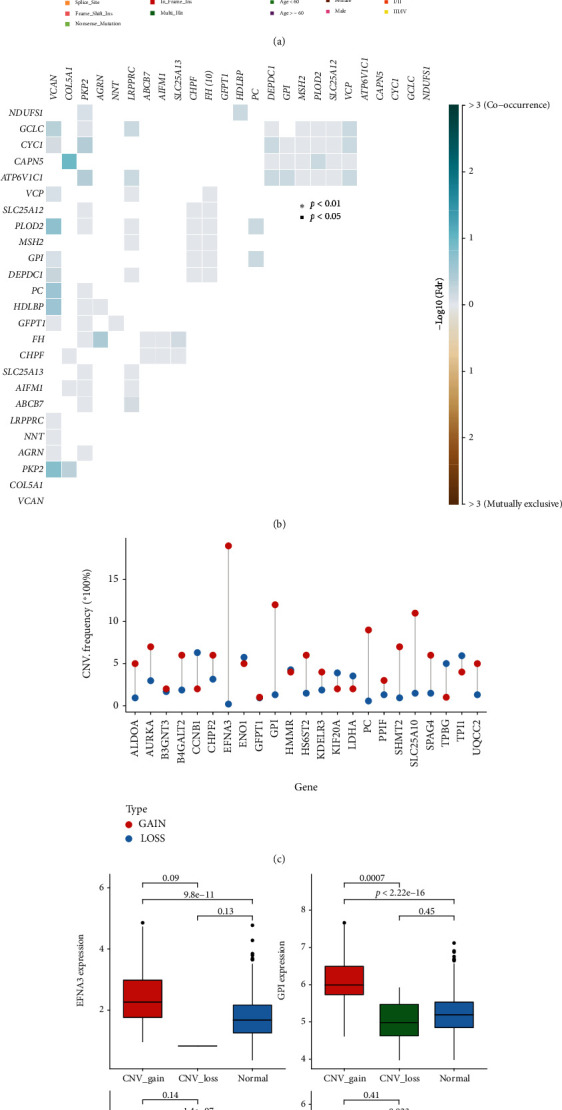
SNV and CNV analysis based on MEMP key genes. (a) TCGA-LUAD patient waterfall plots showing the mutation landscapes of MEMP key genes. (b) Comutation analysis of top25 with the highest mutation frequency in MEMP gene. (c) The majority of MEMP-related genes were amplified in the TCGA-LUAD patients. (d) Boxplots indicating the copy number amplification and the normal had significant differences of MEMP key genes (EFNA3, GPI, SLC25A10, and PC).

The majority of the mutations in the MEMP core gene were cytosine, indicating that this nucleoside is extremely unstable and may indeed be its amino group that was quickly oxidized (Figure 2(a)). Likewise, the overall mutation rate of key MEMP genes was not high. The highest mutation rate of the remaining genes, except VCAN, was just 6%, while the majority had mutation rates around 2%. For comutation analysis, the top 25 MEMP genes with the highest mutation frequency were selected. The results showed that there were no significant cooccurrence and mutual exclusion between MEMP genes with top25 mutations (Figure 2(b)). To display the percentage of copy number amplified and deleted samples in the total sample, the top 25 genes that were significantly upregulated in the MEMP gene (Log2FC > 1, *P* value < 0.05 after correction) were chosen (Figure 2(c)). It can be seen that EFNA3, GPI, SLC25A10, and PC have the most gain. The differences between the four genes in copy number amplification, copy number deletion, and normal copy number were further compared (Figure 2(d)). The outcomes demonstrated that the copy number amplification and the normal had noticeable differences. In conclusion, the MEMP key gene exhibited SNV and CNV variations in normal and cancerous LUAD, further demonstrating the potential association between the MEMP key gene and LUAD.

### 3.3. Construction of MEMP Score Prognostic Model

**Figure 3:**
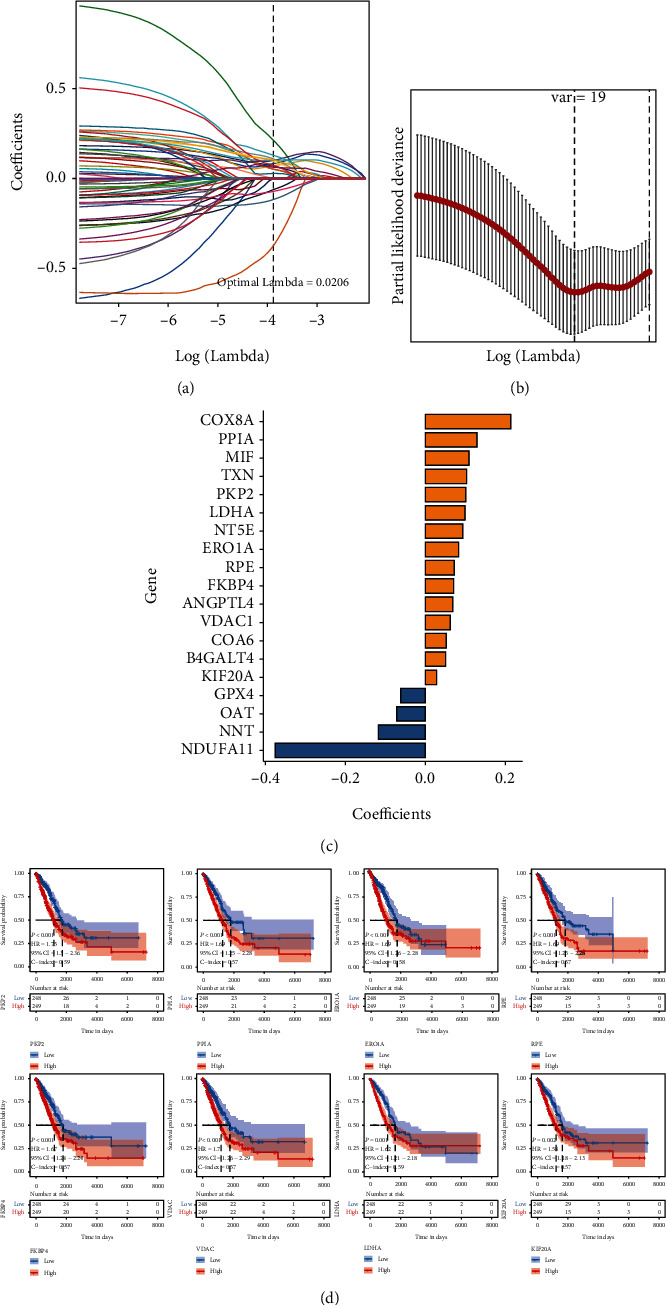
Signature filtering of the MEMP score prognostic model. (a) Change track of each independent variable, the horizontal axis represents the log value of the independent variable lambda, and the vertical axis represents the coefficient of the independent variable. (b) Confidence interval under each lambda. (c) Regression coefficient of the signature. (d) Signature gene of top 8 genes.

266 MEMP key genes were subjected to the univariate Cox regression analysis, which demonstrated that 61 genes had significant relationships with OS, of which 56 genes were high-risk genes (HR > 1, *P* value < 0.05), and their high expression would affect the prognosis of patients. Five genes were low-risk genes (HR < 1, *P* value < 0.05), and their high expression would lower the prognosis. The LASSO-Cox modeling was performed with 61 prognosis-related genes. The value selected 19 genes to constitute the signature and established a prognostic model (Figures 3(a) and 3(b)). The regression coefficients of 19 genes revealed that 15 of them were high-risk genes with coefficients greater than 0, and 4 were low-risk genes with coefficients less than 0 (Figure 3(c)). The Kaplan-Meier curves were used to evaluate the prognostic values of the top eight genes and showed that the low expression of these eight genes indicated higher survival probability (Figure 3(d)).

**Figure 4:**
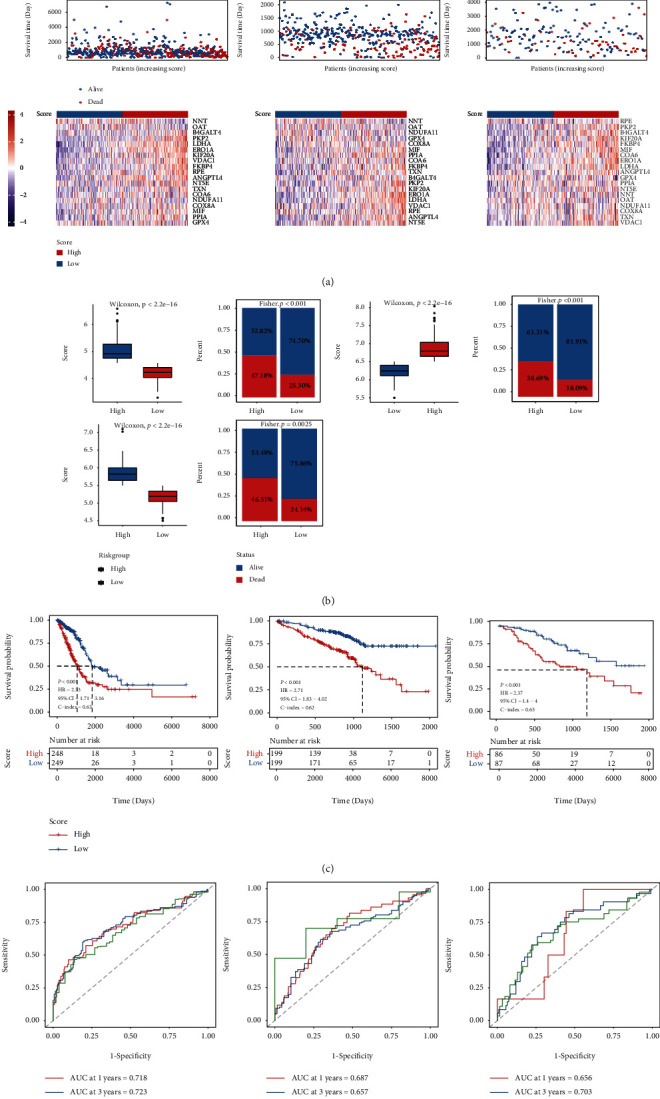
Construction of the MEMP score prognostic model. (a) Score distribution, survival rates, and expression heat maps for high and low score groups in TCGA, GSE72094, and GSE43127, respectively. (b) Score variation between groups with high and low scores as well as the survival and mortality rates in three datasets. (c) Survival of the high and low score groups of TCGA, GSE72094, and GSE43127. (d) Time-dependent ROC curves of TCGA, GSE72094, and GSE43127.

The present study used 19 signature genes to generate the MEMP score for each patient, dividing the patients into high- and low-risk groups, and assessed the differences in gene expression, survival, and distribution between the two groups (Figures 4(a) and 4(b)). The findings demonstrated that there were distributional differences between high- and low-risk groups. Additionally, K-M survival analysis revealed that the high-risk groups' survival probability was much lower than that of the low-risk group (Figure 4(c)). This suggested that for LUAD patients, the MEMP score might be a significant predictive factor.

Utilizing ROC analysis, the survival rates of LUAD at 1, 3, and 5 years were predicted, and the MEMP score was examined as a prognostic predictor. It is evident that the MEMP score may be used to predict 1-, 3-, and 5-year survival rates of LUAD patients (AUC 1 year = 0.718, AUC 3 year = 0.723, and AUC 5 year = 0.687) (Figure 4(d)). Furthermore, the aforementioned findings were validated using GSE72094 and GSE42127. The outcomes also demonstrated the predictive value of MEMP score for LUAD patients, as well as its accuracy in predicting 1-, 3-, and 5-year survival rates.

**Figure 5:**
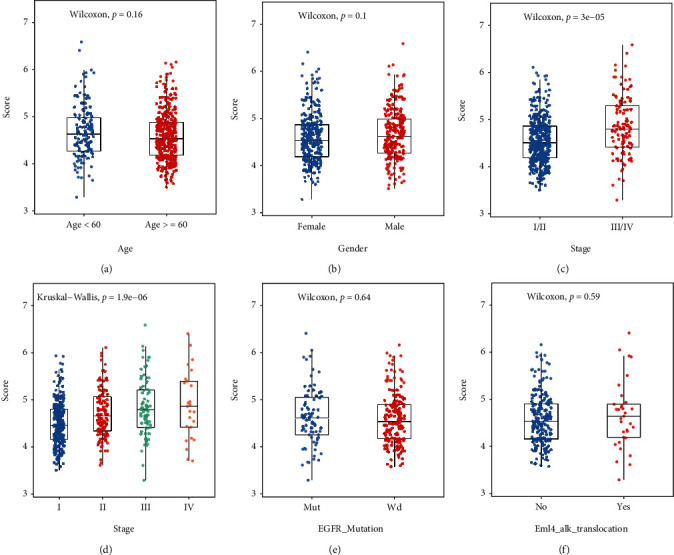
Differences in scores among subgroups with different clinical characteristics. (a–f) Differences in MEMP scores between subgroups with different clinical characteristics (age (a), gender (b), stage (I/II-III/IV) (c), stage (I-IV) (d), EGFR mutation (e), and EML4-ALK translocation (f)), respectively.

**Figure 6:**
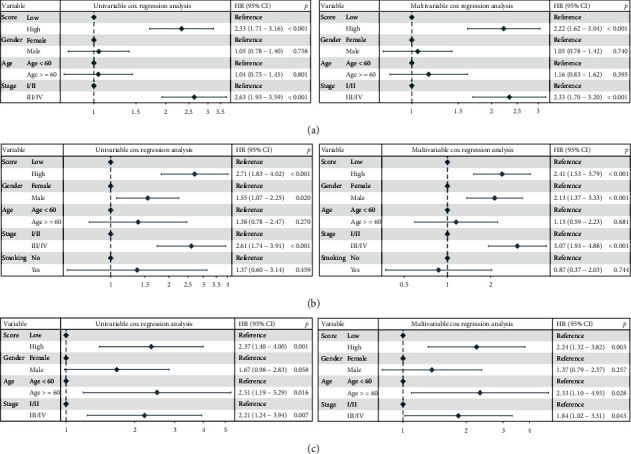
Independent prognostic validation of score in training and validation datasets. (a–c) MEMP scores were an independent prognostic factor for LUAD patients in the TCGA-LUAD (a), GSE72094 dataset (b), and GSE42127 dataset (c).

To identify how MEMP scores vary by clinical features in LUAD patients, the distribution of MEMP scores was compared, including age, gender, stage (I/II-III/IV), stage (I-IV), EGFR mutation, and EML4-ALK translocation (Figures 5(a)–5(f)). It was discovered that there was a substantial difference in scores between patients with TNM stage I/II and stage III/IV, indicating that patients with early cancer and those with late cancer had different risk scores (Figures 5(a)–5(f)). Afterwards, it was determined whether the MEMP score was an independent prognostic factor for LUAD patients by performing univariate and multivariate Cox risk regression analyses (Figures 6(a)–6(c)). The MEMP score and stage were predictive factors associated with LUAD OS, according to a univariate Cox analysis, and they remained significant after multivariate Cox correction.

### 3.4. Construction of Prognostic Nomograms

**Figure 7:**
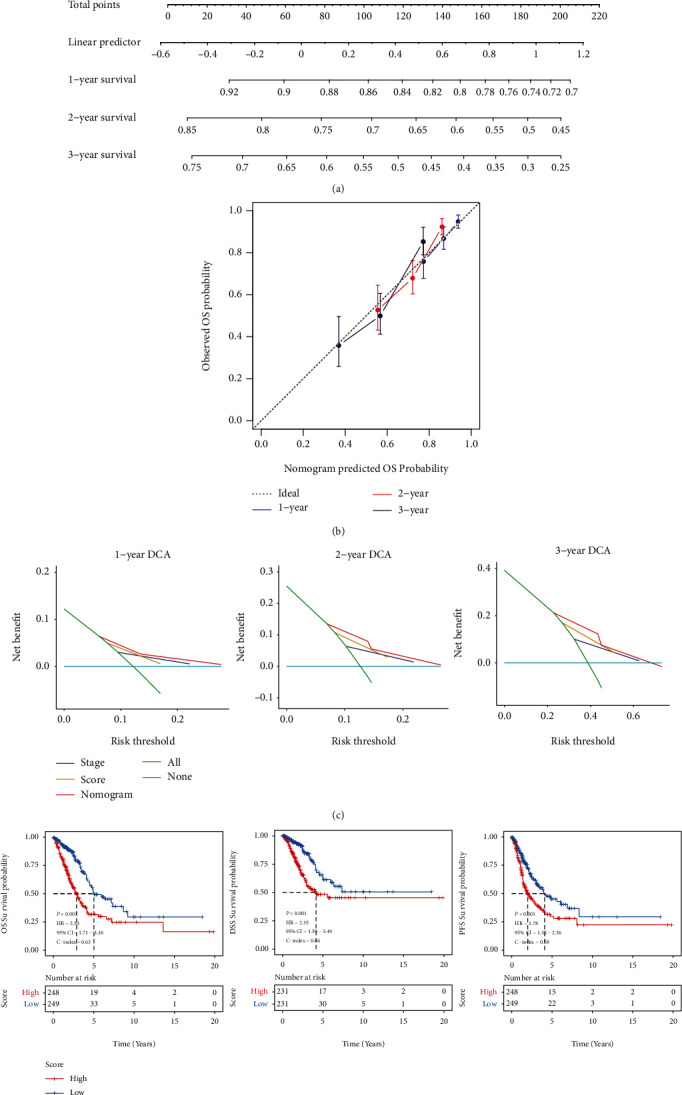
Nomogram construction. (a) Nomograms of LUAD patients. (b) Correction curves for 1-year, 2-year, and 3-year OS survival. (c) DCA decision curve revealing the correlation of net benefits of the TNM stage, MEMP score, nomogram, and all three for OS survival at 1, 2, and 3 years. (d) Survival of OS, DSS, and PFS in high- and low-risk groups of patients with lung adenocarcinoma.

The MEMP score and stage were additionally used to create a prediction nomogram for forecasting the 1-, 2-, and 3-year OS survival rates in order to establish a therapeutically useful, steady, and reliable prediction model for patients with LUAD (Figure 7(a)). The calibration curve suggested that the nomogram was accurate in predicting the 1-year, 2-year, and 3-year OS rates of patients with LUAD (Figure 7(b)). Decision curve analysis (DCA) demonstrated that the nomogram composite model had a better impact than any single independent predictive factor of stage and score (Figure 7(c)). These results showed that the MEMP score combined with the patient stage can accurately predict the 1-year, 2-year, and 3-year survival rates of patients with LUAD and can provide a prediction model. Furthermore, the prognostic significance of the nomogram, which was observed to be strongly correlated with OS, DSS, and PFS was validated, respectively (Figure 7(d)).

### 3.5. MEMP Score Associated Immune Infiltration and Immunotherapy

**Figure 8:**
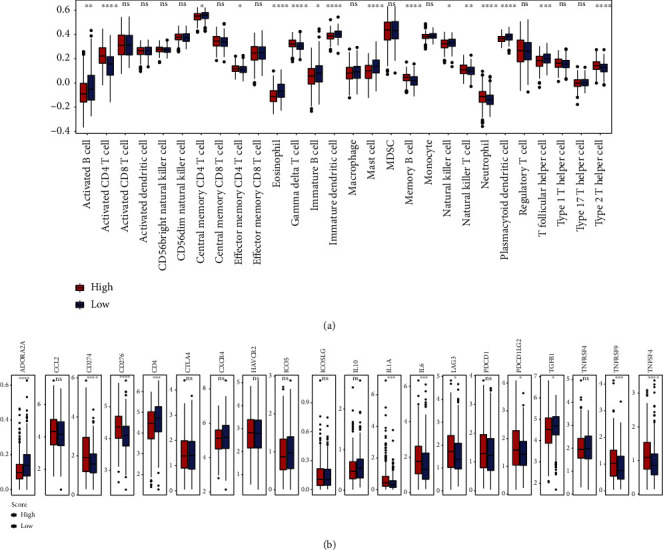
MEMP score associated immune infiltration and treatment. (a) Correlation of cell infiltration level in tumor microenvironment between high and low by ssGSEA. (b) Differences of immune checkpoint expression in high-risk and low-risk groups.

Based on TCGA-LUAD transcriptome data, the degree of immune infiltration across groups with high and low MEMP scores was examined for further understanding of the potential mechanisms (Figure 8(a)). According to ssGSEA, immune infiltration level was higher in the high-risk score group for activated CD4 T cells, Gamma delta T cells, and type 2 T helper cells, while it was higher in the low-risk group for eosinophils, mast cells, immature B cells, and immature dendritic cells.

Since the immune checkpoint expression is a crucial indicator of the individualized immunotherapy, the differences between immune checkpoint expression in the high and low-risk score groups were further analyzed (Figure 8(b)). It was found that the expression levels of ADORA2A, CD4, and TGFB1 were higher in the low-risk population, but the expression levels of CD274, CD276, IL1A, LAG3, PDCD1LG2, TNFRSF9, and TNFSF4 were higher in the high-risk population. In the high-risk group, there was a noticeably higher expression of the majority of immunological checkpoints. This might be connected to the benefits of immunotherapy for those with tumors. For instance, CD274 (PD-L1), which is linked to immune system suppression, was expressed at much higher levels in the high-risk group.

### 3.6. Prediction of Potential Therapeutic Drugs

**Figure 9:**
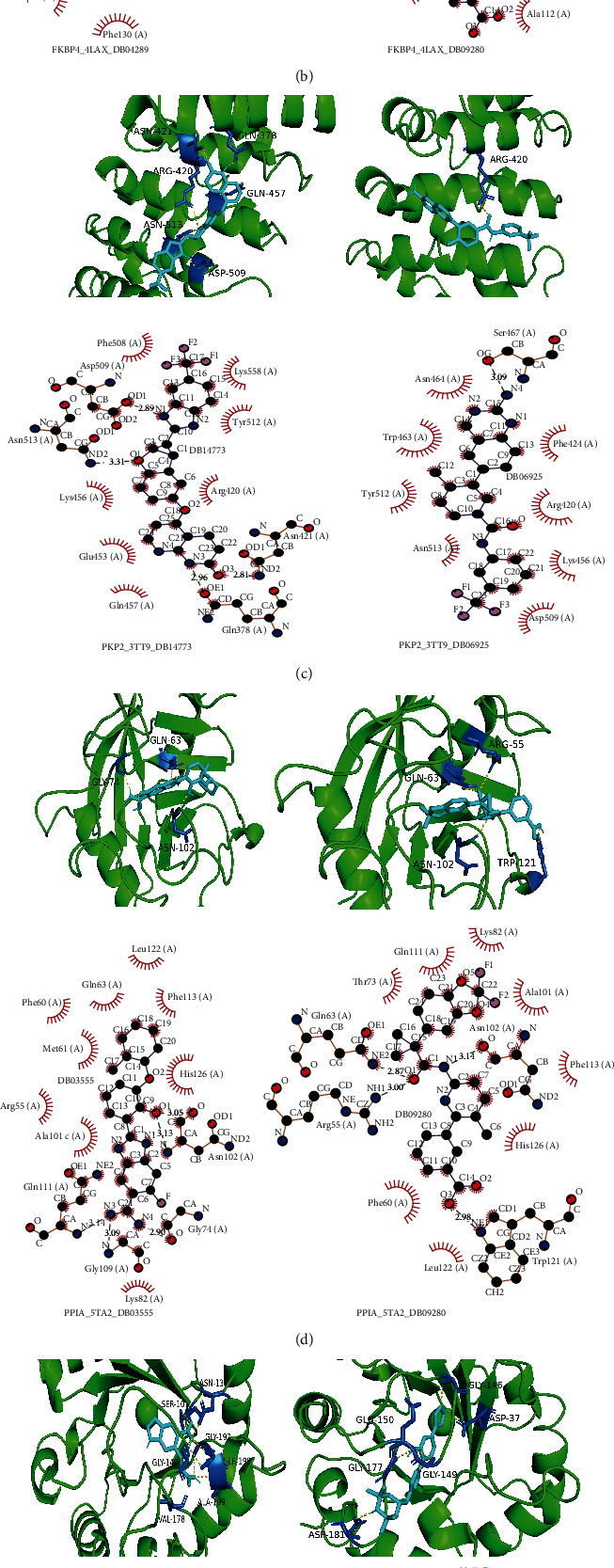
Protein molecular docking and interaction analysis. (a–f) Top 2 interaction conformation and interaction of 6 MEMP key proteins (ERO1A (a), FKBP4 (b), PKP2 (c), PPIA (d), RPE (e), and VDAC1 (f)), respectively.

The MEMP score prognostic model had a strong predictive efficiency, and the MEMP score significantly connected with the prognosis of LUAD patients and immune cell infiltration in TME. Therefore, molecular docking was employed to further explore prospective therapeutic drugs for LUAD using the MEMP signature genes. The related compound structure data retrieved from the DrugBank database were screened according to Lipinski's criteria, and 5464 small molecule compounds were ultimately obtained. As potential therapeutic targets, the top six prognostic important genes, ERO1A, FKBP4, PKP2, PPIA, RPE, and VDAC1, were chosen, whose 3D structures were acquired from the PDB database. Six candidate genes were docked with small molecule compounds using AutoDock Vina, the interaction forces were examined by Pymol and Ligplus, and the top two small molecules with the highest interaction force scores were selected for display (Figures 9(a)–9(f)). It can be seen that the six MEMP signature proteins interacted strongly and closely with the appropriate small molecule compounds, suggesting that these compounds may be developed for the treatment of LUAD. Among them, there were strong interactions existing between DB09080 and FKBP4 and PPIA. The results of the clinical trials demonstrated that the administration of orkambi (lumacaftor/ivacaftor), a known mature medicine, can improve lung function and reduce the likelihood of lung deterioration. Therefore, it is likely that DB0980 will be used as a medication to treat lung adenocarcinoma.

## 4. Discussion

It is crucial to discover effective treatment options for LUAD due to the complex pathophysiology of the disease [13]. In recent years, radiotherapy, chemotherapy, immunotherapy, target medication therapy, and thoracic surgery have all been used as LUAD therapeutic options [2, 14]. Although there has been some improvement, the patient prognosis remains challenging. This may be due to a lack of a comprehensive understanding of the underlying mechanisms of LUAD. Additionally, MEMP processes like glycolysis and oxidative phosphorylation play a significant role in the development and occurrence of malignancies and may independently affect the prognosis for LUAD patients [15–18]. Therefore, it is necessary and urgent to comprehend the probable mechanisms of LUAD in MEMP-related functions, investigate the elements that affect the prognosis of LUAD, and create novel and powerful prognostic features.

In this study, TCGA-LUAD data, combined with the gene set related to MEMP, were used to obtain five gene sets, which were significantly related to LUAD by GSEA analysis. These gene sets included one associated with glycolysis, three associated with oxidative phosphorylation, and one associated with the ATP electron transport chain. Additionally, 266 key MEMP genes were further screened. A new predictive characteristic of LUAD called the MEMP score was created using 19 key MEMP genes identified by LASSO-Cox regression. As an accurate independent predictive factor for LUAD, the MEMP score has been effectively replicated in the validation set. It also has strong predictive efficacy for LUAD patients. Based on LUAD TNM staging, it is also possible to predict the 1-year, 2-year, and 3-year survival rates of LUAD patients. To clarify the potential correlation, the ssGSEA was used to calculate the abundance of distinct immune cells in each LUAD sample. Activated CD4 T cell, type 2 T helper cell, Gamma delta T cell, and other immune cells have a better immune infiltration effect in the high-risk group. However, eosinophil, mast cell, immaturity B cell, and immaturity dendritic cell have better effect in the low-risk group. High-risk individuals had more severely compromised antitumor immunity. Moreover, the acquired features give tumor cells the ability to activate immunological checkpoint pathways that bypass immune surveillance and reduce the immune response. Numerous diseases may develop or worsen because of the aberrant expression of immunological checkpoint markers. Immune checkpoint blockade has recently achieved significant advancements and has established itself as the gold standard for cancer treatment [19]. The majority of high-risk groups have notably higher immunological checkpoint levels, suggesting that high-risk patients have a greater capacity to evade immune surveillance and suppress antitumor immunity. From this vantage point, the prediction model might offer a useful means of accelerating customized cancer immunotherapy.

Effective targeted drug therapy is one of the commonly used methods in tumor therapy. Through molecular docking analysis, 5464 small molecules interacting with signature protein were found. These molecules may be used as potential therapeutic drugs, providing ideas and resources for future research on targeted drugs for LUAD. Six signature genes with the strongest correlation with the prognosis of LUAD were selected, and the two small molecules with the highest association of each gene were obtained through protein interaction. 11 small compounds that can simultaneously and closely bind to DB09080, FKBP4, and PPIA were discovered. It is found in the DrugBank database that three small molecules are related to drug research, including DB09080 (lumacaftor), DB09280, and DB14773. DB09280 (lumacaftor) is a medication that is combined with ivacaftor and may be used to treat cystic fibrosis (CF) in people six years of age and older. DB14773 is being investigated for the treatment of locally progressed or metastatic malignant tumors (clinical trial: NCT03641586), while DB14918 is being studied among patients with spinal muscular atrophy (SMA) (clinical trial: NCT02268552) [20]. According to the outcomes of DB09080 clinical trials, the use of orkambi (lumacaftor/ivacaftor) can enhance lung health and reduce the risk of lung deterioration [21]. DB0980 will therefore probably be utilized as a drug to treat LUAD. Although more experimental evidence is required for these medicinal molecules, this study can increase the probability that drugs will be developed successfully.

There are still certain restrictions on the current investigation. The effect of independent prediction of LUAD prognosis by the MEMP score is still limited. In order to predict the prognosis of LUAD in conjunction with MEMP score in the future, it is required to integrate further prognostic variables. This could increase the prediction accuracy of the model for LUAD patient prognosis. Further research is required to confirm the link between the MEMP score and the immunotherapy response. In this respect, the main limitation of the current study is that it is restricted on a theoretical model, while no clinical validation of the results was performed. In this context, all bioinformatics studies are limited; however, they can provide a basis for subsequent clinical research. Meanwhile, bioinformatics analysis is an appropriate method do derive hypotheses from available data as a basis for future translational research. Therefore, also the current study's findings must be interpreted as a hypothesis, needing clinical validation.

## 5. Conclusions

This current study developed the MEMP score as a new independent prognostic feature of LUAD to predict survival. The findings underline the importance of mitochondrial energy metabolism pathway in LUAD. The small molecule DB0980 was considered to be the most likely drug for the treatment of LUAD.

## Figures and Tables

**Table 1:** Clinical phenotype of TCGA-LUAD.

Phenotype	Group	No. (%)
Age	Age < 60	136 (27.36%)
	Age ≥ 60	351 (70.62%)
	Unknown	10 (2.02%)
Gender	Female	269 (54.12%)
	Male	228 (45.88%)
Stage	Stage I	267 (53.72%)
	Stage II	118 (23.74%)
	Stage III	80 (16.1%)
	Stage IV	25 (5.03%)
	Unknown	7 (1.41%)
EGFR mutation	Mut	79 (15.9%)
	Wd	190 (38.23%)
	Unknown	228 (45.87%)
EML4-ALK translocation	Yes	33 (6.64%)
	No	206 (41.45%)
	Unknown	258 (51.91%)

**Table 2:** Key genes of the mitochondrial energy metabolism pathway.

Pathway	No.
HALLMARK_GLYCOLYSIS	100
HALLMARK_OXIDATIVE_PHOSPHORYLATION	127
GOBP_OXIDATIVE_PHOSPHORYLATION	95
GOBP_ATP_SYNTHESIS_COUPLED_ELECTRON_TRANSPORT	66
KEGG_OXIDATIVE_PHOSPHORYLATION	78

## Data Availability

The data used to support the findings of this study are available from the corresponding author upon reasonable request.
